# Impaired cognitive flexibility and disrupted cognitive cerebellum in degenerative cerebellar ataxias

**DOI:** 10.1093/braincomms/fcae064

**Published:** 2024-02-23

**Authors:** Jung Hwan Shin, Heejung Kim, So Yeon Lee, Won Tae Yoon, Sun-Won Park, Sangmin Park, Dallah Yoo, Jee-Young Lee

**Affiliations:** 1 Department of Neurology, Seoul Metropolitan Government-Seoul National University Boramae Medical Center and Seoul National University College of Medicine, Seoul 07061, South Korea; Department of Neurology, Seoul National University Hospital and Seoul National University College of Medicine, Seoul 03080, South Korea; Department of Nuclear Medicine, Seoul Metropolitan Government-Seoul National University Boramae Medical Center, Seoul 07061, South Korea; Department of Neurology, Institute of Radiation Medicine, Medical Research Center, Seoul National University, Seoul 03080, South Korea; 1 Department of Neurology, Seoul Metropolitan Government-Seoul National University Boramae Medical Center and Seoul National University College of Medicine, Seoul 07061, South Korea; Department of Neurology, Kangbuk Samsung Hospital, Sungkyunkwan University School of Medicine, Seoul 03181, South Korea; Department of Radiology, Seoul Metropolitan Government-Seoul National University Boramae Medical Center and Seoul National University College of Medicine, Seoul 07061, South Korea; Department of Radiology, Seoul National University College of Medicine, Seoul 03080, South Korea; Department of Neurology, Eulji University Hospital, Eulji University School of Medicine, Daejeon 35233, South Korea; Department of Neurology, Kyung Hee University College of Medicine, Kyung Hee University Hospital, Seoul 05278, South Korea; 1 Department of Neurology, Seoul Metropolitan Government-Seoul National University Boramae Medical Center and Seoul National University College of Medicine, Seoul 07061, South Korea

**Keywords:** cognitive flexibility, Wisconsin Card Sorting Test, degenerative cerebellar ataxia, clinical biomarker, cognitive cerebellum

## Abstract

There is a clinically unmet need for a neuropsychological tool that reflects the pathophysiology of cognitive dysfunction in cerebellar degeneration. We investigated cognitive flexibility in degenerative cerebellar ataxia patients and aim to identify the pathophysiological correlates of cognitive dysfunction in relation to cerebellar cognitive circuits. We prospectively enrolled degenerative cerebellar ataxia patients with age-matched healthy controls who underwent 3 T 3D and resting-state functional MRI. All 56 participants were evaluated with the Scale for Assessment and Rating of Ataxia and neuropsychological tests including the Wisconsin Card Sorting Test, Trail Making Test, Montreal Cognitive Assessment and Mini-Mental State Examination. From MRI scans, we analysed the correlation of whole-brain volume and cortico–cerebellar functional connectivity with the Wisconsin Card Sorting Test performances. A total of 52 participants (29 ataxia patients and 23 healthy controls) were enrolled in this study. The Wisconsin Card Sorting Test scores (total error percentage, perseverative error percentage, non-perseverative error percentage and categories completed), Trail Making Test A and Montreal Cognitive Assessment were significantly impaired in ataxia patients (*P* < 0.05) compared to age-matched healthy controls. The Wisconsin Card Sorting Test error scores showed a significant correlation with the ataxia score (*P* < 0.05) controlling for age and sex. In volumetric analysis, the cerebellar right crus I, II, VIIb and VIII atrophy correlated with non-perseverative error percentage in the ataxia group. In functional connectivity analysis, the connectivity between crus I, II and VIIb of the cerebellum and bilateral superior parietal and superior temporal gyrus was significantly altered in ataxia patients. The functional connectivity between left crus II and VIIb of the cerebellum and dorsolateral prefrontal and superior frontal/parietal cortices showed a positive correlation with perseverative error percentage. The connectivity between left crus VIIb and pontine nucleus/middle cerebellar peduncle showed a significant negative correlation with non-perseverative error percentage in the ataxia group. The impaired cognitive flexibility represented by the Wisconsin Card Sorting Test was significantly impaired in degenerative cerebellar ataxia patients and correlated with disease severity. The Wisconsin Card Sorting Test performance reflects hypoactivity of the cognitive cerebellum and disrupted cortico–cerebellar connectivity in non-demented patients with degenerative cerebellar ataxia.

## Teaser

Shin *et al*. examined cognitive flexibility in degenerative cerebellar ataxia patients through the Wisconsin Card Sorting Test (WCST), which was significantly impaired in correlation with the severity of ataxia. The performance on the WCST was correlated with atrophy of the cognitive cerebellum and disruptions in cortico–cerebellar connectivity in patients with degenerative cerebellar ataxia.

## Graphical Abstract

**Graphical Abstract:**
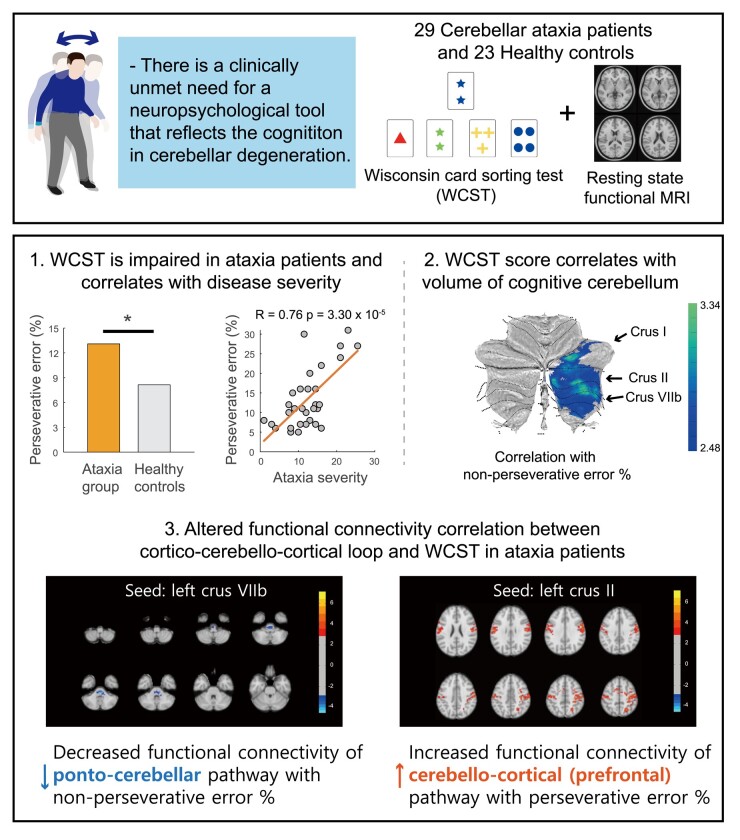

## Introduction

The cerebellum plays a crucial role in coordinating motor programmes, and disruptions in cerebellar circuits can lead to various motor impairments, including oculomotor disturbances, speech deficits, limb dysmetria and postural imbalance. In addition, clinical studies have revealed that cerebellar disease patients also exhibit neuropsychological impairments, primarily involving attentional/frontal executive dysfunction, as well as visuospatial and memory dysfunction.^1-4^ These unique spectra of cognitive impairment in cerebellar patients have been observed in different disease types including degenerative cerebellar ataxias (CAs).^2,5-8^

The underlying pathophysiological mechanism of cognitive dysfunction in cerebellar disease has been investigated with anatomical, physiological and functional imaging studies.^9,10^ These studies revealed a topographic organization of the cognitive cerebellum that is mainly composed of crus I, crus II and lobule VIIb.^11^ The cognitive cerebellum has reciprocal feedforward and feedback connections with the prefrontal, posterior parietal, superior temporal or cingulate gyrus without connection with the sensorimotor cortex or spinocerebellar tract.^12-14^ The cognitive task-related functional MRI (fMRI) and PET studies revealed activation of the cognitive cerebellum crus I and II in visuospatial processing and working memory,^15,16^ crus I and lobule VIIb in frontal executive^10,17^ and crus I and lobule VI in language and verbal memory functions.^9,10^ Furthermore, imaging studies also revealed simultaneous activation of prefrontal and parietal cortices that have a reciprocal connection with the cognitive cerebellum during the corresponding cognitive tasks.^16^ These observations suggest the cortico–cerebello–thalamo–cortical loop as the cognitive circuit of the cerebellum.^12^

However, the cognitive dysfunction in patients with cerebellar degeneration may not be easy to identify in clinical practice as the cognitive symptoms are often neglected and a comprehensive neuropsychological battery, especially focused on frontal/executive function, is required to characterize the unique feature of cerebellar cognitive dysfunction.^17^ As a result, cognitive dysfunction in cerebellar degeneration is sometimes underdiagnosed or underestimated.^2,18,19^ Therefore, there is a clinically unmet need for an accessible neuropsychological tool that reflects the underlying pathophysiology of cognitive dysfunction and correlates with the disease severity of cerebellar degeneration in progressive CAs.

Cognitive flexibility, which refers to the ability to adjust to a shifting set, has been reported to be impaired in patients with cerebellar degeneration.^20^ Also, a correlation of cognitive flexibility with cerebellar lobular volume alteration has been found in neuropsychiatric disorders including addiction, autism, schizophrenia, attention-deficit/hyperactivity disorders and obsessive-compulsive disorders.^21^ The Wisconsin Card Sorting Test (WCST) is a commonly used instrument for evaluating cognitive flexibility.^22^ Although a few studies have examined the performance of WCST in patients with degenerative CA,^23-25^ the clinical and structural correlations of WCST performance in this population have not been clearly elucidated.

From this perspective, we hypothesized that performance in cognitive flexibility may reflect the degeneration in the cognitive part of the cerebellum, and be a useful tool to evaluate cognitive function in patients with degenerative CA. To investigate this hypothesis, we conducted a case–control study in which we utilized the WCST and examined the correlation between impairment in cognitive flexibility and the severity of ataxia, volume of cerebellar atrophy and alterations in the functional connectivity (FC) of cortico–cerebellar pathways in patients with degenerative CA.

## Materials and methods

### Study participants

We prospectively enrolled patients with insidious adult onset (>age 30) primary degenerative CA from the neurology clinic at the Seoul National University Boramae Medical Center (SNU-BMC) between 2018 and 2020. The inclusion criteria consisted of age of onset above 30 years and a diagnosis of either hereditary spinocerebellar ataxias (SCAs) or idiopathic late-onset CAs. Patients with additional clinical features including severe autonomic dysfunction, parkinsonism/dystonia or dementia were excluded. Multiple system atrophy and secondary causes for CA including inflammatory, infectious, paraneoplastic, vascular, toxic and structural lesions were also excluded. Age-matched healthy controls (HCs) were recruited during the same study period at SNU-BMC. The Institutional Review Board of SNU-BMC approved this study (IRB No. 20-2018-90), and written informed consent was obtained from every participant.

### Clinical evaluation

Baseline demographic and clinical information was recorded in every participant including family history. We evaluated the severity of ataxia with the Scale for Assessment and Rating of Ataxia (SARA). The cognitive function was evaluated with the WCST (Computer Version 4-Research Edition, PAR), K-Mini-Mental State Examination (MMSE), Montreal Cognitive Assessment-K (MoCA), Trail Making Test (TMT) A and B, and K-Beck’s Depression Inventory. For the WCST performance, total error (raw score and percentage), perseverative error (raw score and percentage), non-perseverative error (raw score and percentage) and categories completed scores were extracted and analysed.

### Imaging acquisition and processing

All participants underwent 3T MRI (Philips Achieva scanner, The Netherlands). 3D sagittal T_1_-weighted spoiled gradient recalled MRI sequence (repitition time (TR) = 9.9 ms, echo time (TE) = 4.6 ms, flip angle = 8°, matrix size = 224 × 224 × 180, field of view (FOV) = 220 × 220 mm^2^, slice thickness = 1 mm, voxel size = 0.98 × 0.98 × 1 mm, 224 slices) and T_2_*-weighted gradient echo planar imaging for resting-state fMRI (180 volumes, TR = 2700 ms, TE = 35 ms, flip angle = 90°, matrix size = 144 × 144, FOV = 220 × 220 mm^2^, voxel size = 1.53 × 1.53 × 4 mm, slice thickness = 4 mm, 35 axial planes parallel to the anterior and posterior commissure lines) sequences were acquired and subsequently analysed. For the latter sequence, participants were instructed to remain motionless, to keep their eyes closed and to try thinking of nothing.

### Voxel-based morphometry analysis

Preprocessing and statistical analysis were performed using the Statistical Parametric Mapping (SPM12, Wellcome Department of Imaging Neuroscience, London, UK, http://www.fil.ion.ucl.ac.uk/spm) implemented in MATLAB 9.12 (The MathWorks, Inc., Natick, MA, USA). Volumetric analysis was performed using the Computational Anatomy Toolbox (CAT12, https://neuro-jena.github.io/cat) embedded in SPM12 to identify structural changes. Each image was segmented into grey matter (GM), white matter (WM) and CSF and nonlinearly normalized to a standard stereotactic space using the DARTEL algorithm. The spatially normalized images were then rescaled to preserve relative tissue volumes and smoothed using 8 mm full width at half maximum (FWHM) Gaussian kernel. For the exclusion of artefacts on the GM, an absolute threshold of 0.1 was applied.

### Spatially Unbiased Infratentorial Template analysis

Individual MRI data were preprocessed and analysed using the Spatially Unbiased Infratentorial Template (SUIT) toolbox (http://www.diedrichsenlab.org/imaging/suit.htm).^26,27^ This tool could achieve more accurate cerebellum inter-subject alignment than the whole-brain methods. The anatomical image was cropped to isolate the cerebellum. The isolated images were segmented into GM, WM and CSF. The segmented GM images were normalized into the cerebellar template provided by SUIT. The GM maps were then smoothed with a 4 mm FWHM Gaussian kernel.

### FC analysis

Functional resting images were preprocessed using DPABI (Data Processing and Analysis for Brain Imaging, http://dpabi.org) based on MATLAB. For each subject, the first 10 volumes were discarded to allow signal stabilization. The remaining 170 volumes were subjected to slice timing correction and head motion correction by realigning with a six-parameter spatial transformation to correct for geometrical displacement. Data with head motion exceeding 2 mm were excluded from this analysis. Following motion correction, data were spatially normalized into MNI space using the standard echo planar imaging template and smoothed with a 4 mm FWHM Gaussian kernel. Then the images were preprocessed by regressing out Friston’s 24-motion parameters, CSF and WM signals as nuisances. Finally, temporal band-pass filtering (0.01–0.08 Hz) was performed on those images. For FC analysis, we set seed regions as bilateral 7b, crus1 and crus2 of the cerebellum using the AAL atlas. For extracting the time course signal of seed regions of interest (ROIs), we used the average of the time series of all voxels in the ROI. Then the correlation coefficient between the average time course of the seed and that of the whole brain was computed in a voxel-wise manner. The coefficients were converted to *Z*-values using Fisher’s *R*-to-*Z* transformation.

### Statistical analysis

We employed independent *t*-tests to compare the mean between the groups. To compare cognitive variables, including WCST, between CA patients and controls, we utilized a general linear model with age and sex as covariates. Additionally, when conducting correlation analysis between cognitive variables and SARA scores, we employed partial correlation analysis, adjusting for age and sex. In the correlation analysis of clinical and cognitive parameters with whole-brain voxel-based morphometry (VBM) and SUIT VBM, we included age, sex and total intracranial volume as covariates. For the correlation analysis of clinical and cognitive parameters with FC, we included age and sex as covariates. In multiple corrections, a Gaussian random field (GRF) model was applied in imaging analysis, including whole-brain VBM, SUIT VBM and FC. False discovery rate was used in the analysis of correlation between clinical parameters. All statistical analysis was performed using MATLAB 2020a (MathWorks, Natick, MA, USA).

## Results

### Baseline clinical characteristics

A total of 52 participants (29 CAs and 23 HCs) were enrolled. The baseline demographic and clinical characteristics are summarized in Table 1. There were no significant differences in age, sex and education years between the CA group and HCs (Table 1).

**Table 1:** Baseline characteristics of the participants

	CA group	Healthy controls	*P*-value
*n*	29	23	
Age	59.34 (8.33)	56.00 (10.13)	0.20
Sex (male/female)	13/16	6/17	0.16
Education years	11.28 (3.46)	11.17 (3.69)	0.91
Disease duration	8.10 (5.99)		NA
SARA	12.37 (5.80)		NA
BDI	16.17 (10.87)	13.50 (12.34)	0.54
MMSE	26.64 (2.80)	27.61 (2.02)	0.24
MoCA	24.51 (2.92)	27.09 (2.62)	0.0098
TMT-A	71.24 (46.48)	33.94 (15.71)	0.015
TMT-B	161.00 (81.06)	115.47 (84.41)	0.27
Total intracranial volume (mL)	1344.20 (137.34)	1420.27 (111.77)	0.038
Total cerebellar volume (mL)	99.03 (13.58)	118.49 (12.15)	3.09 × 10^−4^
Type of CA	19 genetic SCA [SCA1 (2), SCA2 (10), SCA3 (5), SCA6 (1), SCA7 (1)], 10 ILOCA		

Data are shown as the mean (standard deviation) or *n*-values. In comparing age, education years, BDI, total intracranial volume and total cerebellar volume between CA group and HCs, *P*-values were done with independent *t*-test. In the comparison of cognitive profiles between CA group and HCs, *P*-values were calculated with generalized linear model with age and sex as cofactors. CA, cerebellar ataxia; MMSE, K-Mini-Mental State Examination; MoCA, Montreal Cognitive Assessment; TMT, Trail Making Test; BDI, K-Beck’s Depression Inventory; SARA, Scale for Assessment and Rating of Ataxia; SCA, spinocerebellar ataxia; ILOCA, idiopathic late-onset cerebellar ataxia.

### Cognitive profile in patients with degenerative CA and its relationship with ataxia severity

The CA group showed significantly poor performance in MoCA and TMT-A compared with the controls, whereas TMT-B, BDI and MMSE scores did not have a significant difference between the CA group and HCs (Table 1). The baseline clinical and cognitive profiles did not differ between genetically confirmed SCA and idiopathic late-onset cerebellar ataxia (ILOCA) patients (Supplementary Table 1). CA group demonstrated significantly worse performance than HCs in WCST subscores both in raw and percentage (error divided by trials) scores of total error, perseverative error, non-perseverative error, perseverative response and categories completed (Table 2).

**Table 2:** Comparison of performance of WCST between CA group and controls

WCST	CA group	Healthy controls	*P*-value
Total error	26.93 (17.51)	13.21 (4.95)	0.0036
Total error (%)	26.65 (13.46)	15.26 (3.58)	0.0012
Perseverative error	13.17 (8.69)	7.43 (3.49)	0.021
Perseverative error (%)	13.48 (7.47)	8.57 (2.45)	0.015
Non-perseverative error	13.76 (9.42)	5.78 (2.39)	0.0013
Non-perseverative error (%)	13.38 (7.59)	6.70 (2.51)	6.47 × 10^−4^
Categories completed	4.41(2.21)	5.96 (0.21)	0.0081

Data are shown as the mean (standard deviation). *P*-values were calculated with generalized linear model with age and sex as cofactors. WCST, Wisconsin Card Sorting Test; CA, cerebellar ataxia.

**Figure 1:**
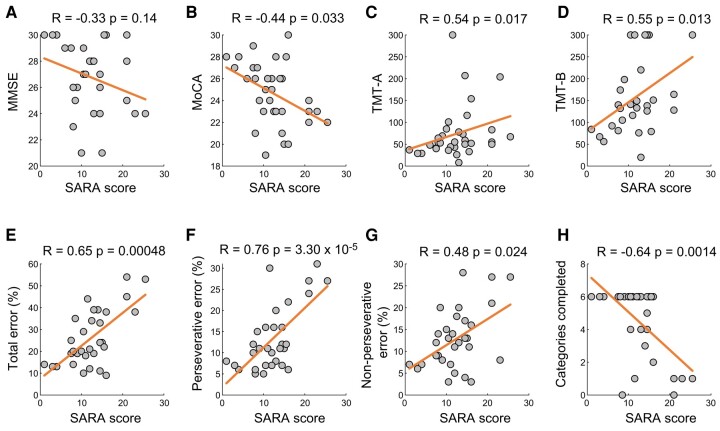
**Correlation of SARA scores with cognitive profiles in CA patients.** Scatter plots of the SARA score with cognitive profiles of MMSE (**A**), MoCA (**B**), TMT-A (**C**), TMT-B (**D**) and WCST scores (**E**-H) in the SCA group. The correlation was analysed with partial correlation with age and sex as cofactors. *R*- and *P*-values are described in each figure. SARA, Scale for Assessment and Rating of Ataxia; TMT, Trail Making Test; MoCA, Montreal Cognitive Assessment; MMSE, Mini-Mental State Examination; WCST, Wisconsin Card Sorting Test; CA, cerebellar ataxia.

MoCA, TMT-A, TMT-B and WCST scores significantly correlated with SARA scores after adjustment for age and sex in CA patients (Fig. 1; Supplementary Fig. 1 for subgroup of CA). In the WCST performance, total error percentage, perseverative error percentage, non-perseverative error percentage scores and categories completed were all significantly correlated with SARA scores (adjusted *P* < 0.05; Fig. 1) whereas the MMSE score did not correlate with SARA scores.

### Correlation of cerebellar volumes with cognitive flexibility and ataxia severity

**Figure 2:**
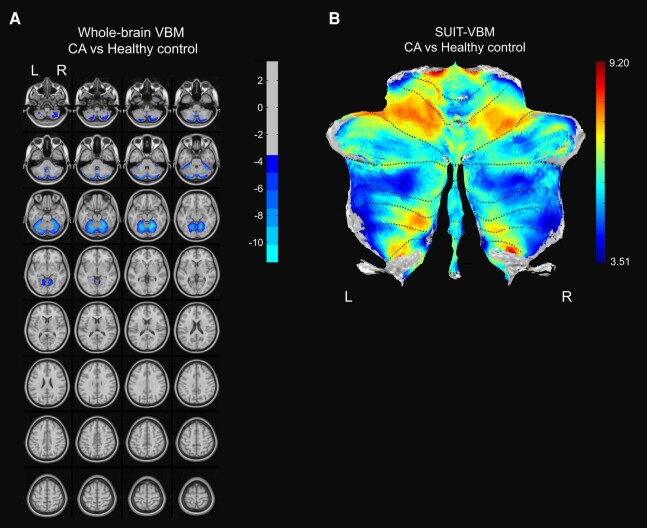
**Comparison of whole-brain and cerebellar volume between CA patients and HCs.** (**A**, **B**) Comparison of whole-brain volume (**A**) and cerebellar volume (**B**) between the CA group and HCs. The comparison was performed with a voxel-wise independent *t*-test. Multiple corrections were done with the GRF method (voxel *P* < 0.001, cluster *P* < 0.01 for whole-brain VBM and voxel *P* < 0.05, cluster *P* < 0.05 for SUIT analysis). The reference bar in **A** and **B** represent the *T*-value. L, left; R, right; VBM, voxel-based morphometry; CA, cerebellar ataxia.

The CA patients showed significantly reduced total cerebellar volume compared with HCs (Table 1). In the whole-brain VBM and SUIT VBM analysis, the CA group showed significantly lower volume in all cerebellar subregions (Fig. 2A and B).

**Figure 3:**
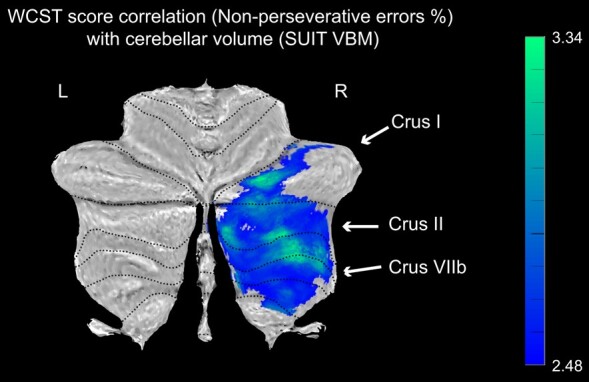
**Significant correlation of cerebellar volume with WCST score in CA patients.** Cerebellar regional correlation with non-perseverative error percentage in WCST. Multiple corrections were done with the GRF method (voxel *P* < 0.05, cluster *P* < 0.05). Colour bar represents *T*-value. CA, cerebellar ataxia; WCST, Wisconsin Card Sorting Test; SUIT: Spatially Unbiased Infratentorial Template; VBM, voxel-based morphometry; L, left; R, right.

Next, we analysed the correlation of cortical GM and cerebellar volumes with WCST scores. In SUIT VBM analysis, the volume of right crus I, crus II, VIIb, VIIIa and VIIIb negatively correlated with non-perseverative error percentage score (Fig. 3). However, there were no significant correlations observed between regional cerebellar volumes and the total error percentage and perseverative error percentage on the WCST. In whole-brain VBM, total error percentage scores negatively correlated with right superior temporal, posterior insular, middle temporal, hippocampal and parahippocampal cortices. Perseverative error percentage scores correlated with right superior temporal, posterior insular, anterior and middle temporal, hippocampal and parahippocampal cortices (Supplementary Fig. 2). The non-perseverative percentage scores did not show any significant correlation. In HCs, we identified negative correlations in right crus I, lobules V and VI and positive correlations in left lobules II and VIIb. With the whole-brain VBM analysis, we found negative correlations of bilateral caudate and putamen volumes with WCST total error percentage, right lingual gyrus and left medial prefrontal volumes with perseverative error percentage and right anterior internal capsule with non-perseverative error percentage (Supplementary Fig. 3).

The SARA scores significantly correlated with total cerebellar volume (partial correlation, *R* = −0.54, *P* = 4.02 × 10^−4^) adjusted for age and sex as cofactors. In SUIT VBM analysis, SARA scores correlated with right crus I (Supplementary Fig. 4).

### Alterations in FC of the cortico–cerebellar loop in correlation with impaired cognitive flexibility

**Figure 4:**
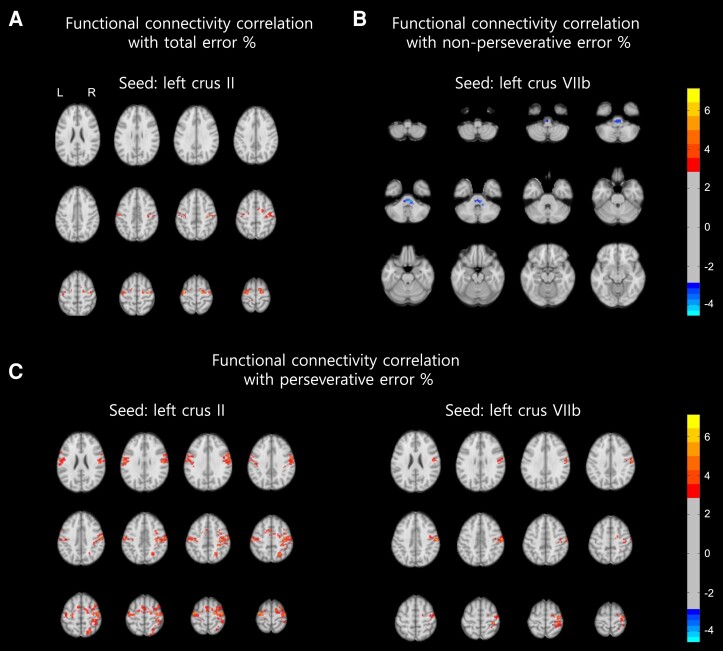
**Correlation of FC of cognitive cerebellum with the performance of WCST in the CA group.** Seed-based whole-brain functional connectomics in the CA group. Multiple corrections were done with the GRF method (voxel *P* < 0.005, cluster *P* < 0.01). Colour bar represents *T*-value. (**A**) FC correlation with total error percentage with left crus II as seed. (**B**) FC correlation with non-perseverative error percentage with left crus VIIb as seed. (**C**) FC correlation with perseverative error percentage with left crus II (left) and VIIb (right) as seeds. Colour bar represents *T*-value. L, left; R, right.

We characterized crus I, II and VIIb of the cerebellum as the cognitive cerebellum based on previous literature, which was also supported by our volumetric analysis (Fig. 3). Then, we analysed whole-brain FC with left and right cognitive cerebellum (crus I, II and VIIb) as the seeds. The CA group showed reduced FC with pons, middle cerebellar peduncle, cerebellar lobule, superior temporal, supramarginal gyrus and superior parietal cortices (Supplementary Fig. 5). In correlation with WCST performance in the CA group, the FC of left crus II with bilateral dorsolateral prefrontal and superior frontal cortices showed a significant positive correlation with total error percentage in the CA group (Fig. 4A). Non-perseverative error percentage showed a negative correlation with FC between left crus VIIb and pontine nucleus/middle cerebellar peduncle (Fig. 4B). The perseverative error percentage score was positively correlated with FC between left crus II, VIIb of the cerebellum, bilateral dorsolateral prefrontal, superior frontal and parietal cortex (Fig 4C).

## Discussion

### Impaired cognitive flexibility in degenerative CA

The WCST scores were found to be significantly worse in the CA group and showed a correlation with the severity of ataxia. However, several studies have reported normal performance on the WCST in cerebellar disease patients.^7,28^ Notably, these studies primarily included patients with cerebellar stroke or cerebellitis, suggesting that their findings may not fully represent the cognitive profiles observed in individuals with degenerative CA.

Higher motor demand can potentially impact the performance of the alternating attention task in cerebellar disease patients.^29^ To address this issue, we utilized a computerized version of the WCST, which requires minimal motor demand. This approach helps to minimize the potential influence of motor dysfunction on the evaluation of cognitive flexibility. Moreover, it further supports the diagnostic utility of the WCST in assessing cognitive dysfunction in individuals with CA.

### Neural correlates of cognitive flexibility in CA: perseverative error

Perseverative error in the WCST is characterized by the occurrence of incorrect responses accompanied by persistent use of the same strategies even after a rule switch. These errors are believed to stem from dysfunctions in cognitive processes such as set switching and error detection. The impairment in set switching is associated with dysfunction in the dorsolateral prefrontal cortex, while the dysfunction in error detection is linked to the anterior cingulate cortex.^30-32^ Damage to the prefrontal cortices resulting from stroke, trauma or lobectomy due to epilepsy is closely associated with a higher occurrence of perseverative errors in the WCST.^33^

Neural substrates related to the WCST have been identified through fMRI^32,34^ and PET^35,36^ studies. These studies have shown activation of the fronto–striatal connections involving the prefrontal, parietal cortices and subcortical structures including caudate, putamen and thalamus.^31,32,34-36^ Notably, studies have also revealed activation of the cerebellum when performing WCST in HCs.^36,37^ However, these studies did not specifically reveal the involvement of subregions of the cerebellum, such as the cognitive cerebellum, in the WCST. In our present study, we demonstrated, for the first time, that the perseverative error score correlated with increased FC between the left cognitive cerebellum (crus I, II and VIIb) and bilateral dorsolateral prefrontal cortex in patients with CA. The cognitive function of the cerebellum relies on its reciprocal connections with the frontal and parietal cortices, thalamus, and other regions within the cerebellum, which form the cerebello–thalamo–cortical loop.^12^ This loop encompasses the cortico–ponto–cerebellar pathway and the cerebello–thalamo–cortical pathways, representing the feedforward and feedback loops, respectively.^38^ The positive correlation observed between the FC of the left cognitive cerebellum (crus II and VIIb) and the bilateral dorsolateral prefrontal cortex with the perseverative error score suggests compensatory activation or recruitment of the feedback loop within the cerebello–thalamo–cortical connection. Our findings align with previous evidence of compensatory activation within the cerebello–thalamo–cortical loop in degenerative CA patients with cognitive dysfunctions.^39,40^

In addition to the cerebellum and prefrontal cortices, our whole-brain volumetric analysis revealed significant negative correlations between the perseverative error percentage of the WCST in the CA group and various brain regions, including the superior temporal, anterior and middle temporal, posterior insular and hippocampal cortices (Supplementary Fig. 2). These results are consistent with previous studies that have demonstrated the involvement of multiple non-cerebellar brain regions, such as the medial frontal gyrus,^35^ inferior parietal lobule,^41^ primary/secondary association visual cortex,^42^ hippocampus or subcortical structures^41^ in the cognitive processes underlying the WCST as the task engages various cognitive functions, including attention, working memory, abstraction and decision-making.

The cerebellar and cerebral volumetric correlation with WCST error resulted in distinct pattern between CA group and HCs (Fig. 2; Supplementary Figs. 2 and 3). In HCs, smaller volumes in the caudate, putamen and medial prefrontal cortices were correlated with total error percentage and perseverative error percentage, respectively, in WCST. This result is in line with previous reports showing that these brain areas are activated during WCST in healthy individuals.^34,37^ In CA group, atrophy of wider brain areas was correlated with WCST errors; thus, it could be inferred that broad range of brain circuits might be recruited for a set-shifting task in individuals with defective cerebellum.

### Neural correlates of cognitive flexibility in CA: non-perseverative error

The non-perseverative error in the WCST refers to errors that are distinct from perseverative errors. Although both types of errors reflect disruptions in frontal executive function, the neural correlates of non-perseverative errors have received relatively less attention. In our study, we found that the non-perseverative error percentage score was significantly correlated with atrophy in the cognitive cerebellum, specifically in the right crus I, II and VIIb. These regions are known to be connected to the frontoparietal network during resting-state conditions.^43^ Furthermore, in the FC analysis, we observed a negative correlation between the non-perseverative error percentage score and the ponto–cerebellar pathway within the cortico–cerebellar loop. Lesions in the ponto–cerebellar tract have previously been associated with impairments in executive function, verbal fluency and verbal learning.^44^ These findings suggest the involvement of the feedforward component of the cerebro–ponto–cerebellar tract in non-perseverative errors, which differs from the functional connections implicated in perseverative errors.

### Asymmetrical involvement of cognitive cerebellum in cognitive flexibility in CA

In our study, we observed an asymmetrical involvement of the cognitive cerebellum in cognitive flexibility among individuals with CA. Specifically, we found reduced FC between the left cognitive cerebellum and both hemispheres of the brain. Additionally, when examining the correlation between FC and WCST scores (total error percentage, perseverative error percentage and non-perseverative error percentage), we found significant correlations only in the left cognitive hemisphere (Fig. 4). Furthermore, right cerebellum was associated with error in WCST scores in the HCs (Supplementary Fig. 3). According to the known connections of the cerebellar hemisphere with the contralateral cerebrum, it is understood that the left cerebellar hemisphere plays a role in frontal executive function and visuospatial function, while the right cerebellar hemisphere is associated with frontal executive function and language function.^45^ This corresponds with our findings, as we observed a significant correlation between FC from the left cognitive cerebellum and WCST scores, which require both prefrontal function and visuospatial function.

### Future perspective

This study was designed as a cross-sectional case–control study with a relatively small number of participants. Thus, it would be important to conduct future longitudinal cohort studies with larger population to investigate whether WCST performance can reflect the longitudinal progression of the disease and cognitive impairment. Then, the population was not assessed using comprehensive neuropsychological profiles. Therefore, a future study incorporating comprehensive neuropsychological evaluations would help clarify the correlation between the cerebellum and its associated circuits with specific cognitive domains. Previous reports have indicated that different subtypes of SCAs exhibit distinct patterns of cerebellar degeneration and may present with different cognitive profiles.^1,24,46^ Furthermore, premanifest SCA patients can display subtle cognitive dysfunction.^47,48^ Studies have reported lower performance in frontal executive function tests, such as Stroop and set shifts, in the premanifest SCA2 group.^49,50^ Therefore, the evolution of cognitive dysfunction and cerebellar atrophy from the premanifest to overt disease stages may vary among different types of SCAs. Lastly, it is worth noting that while the manual version of WCST may pose challenges in clinical practice due to resource and manpower requirements, the computerized WCST used in this study is self-administered and semi-automated, making it easier to apply to patients for both clinical and research purposes.

## Conclusion

The impaired cognitive flexibility represented by the WCST test was significantly impaired in degenerative CA patients and correlated with disease severity. WCST performance reflects hypoactivity of the cognitive cerebellum and disrupted cortico–cerebellar connectivity in non-demented patients with degenerative CA.

## Supplementary Material

fcae064_Supplementary_Data

## Data Availability

The data sets and code generated during the current study are available from the corresponding author upon reasonable request, including reproducibility of research or external validation. Restrictions may be applied to sensitive data for privacy preservation.
