# Supplementary material for: Impaired cognitive flexibility and disrupted cognitive cerebellum in degenerative cerebellar ataxias
Source: Brain Commun. 2024 Feb 23;6(2):fcae064. doi: 10.1093/braincomms/fcae064 (PMC10919478; doi:10.1093/braincomms/fcae064)
Supplement: fcae064_Supplementary_Data [file fcae064_supplementary_data.docx]

**Supplementary Table 1.** **Baseline characteristics of genetic cerebellar ataxia and idiopathic late onset cerebellar ataxia**

|  | SCA group | ILOCA group | P-value |
| --- | --- | --- | --- |
| n | 19 | 10 |  |
| Age | 60.21 (9.34) | 57.7 (6.07) | 0.17 |
| Sex (Male/female) | 8/11 | 5/5 | 0.68 |
| Education year | 10.50 (3.85) | 12.70 (2.11) | 0.17 |
| Disease duration | 8.84 (4.39) | 6.7 (8.37) | 0.37 |
| SARA | 13.69 (6.45) | 10.0 (3.55) | 0.11 |
| BDI | 17.21 (11.70) | 14.2 (9.37) | 0.48 |
| MMSE | 25.63 (3.05) | 26.67 (2.45) | 0.90 |
| MoCA | 24.26 (3.21) | 25.0 (2.36) | 0.68 |
| TMT-A | 83.89 (52.69) | 47.2 (13.77) | 0.06 |
| TMT-B | 184.67 (92.53) | 125.5 (43.24) | 0.14 |
| Total intracranial volume (ml) | 1340.95 (131.59) | 1351.09 (156.89) | 0.48 |
| Total cerebellar volume (ml) | 99.55 (11.56) | 97.91 (17.88) | 0.64 |
| WCST scores |  |  |  |
| Total error | 29.73 (18.42) | 21.6 (15.09) | 0.22 |
| Total error (%) | 29.84 (14.08) | 20.6 (10.23) | 0.066 |
| Perseverative error | 14.53 (8.69) | 10.6 (6.50) | 0.22 |
| Perseverative error (%) | 15.16 (8.32) | 10.3 (4.24) | 0.065 |
| Nonperseverative error | 15.21 (9.65) | 11.0 (8.78) | 0.26 |
| Nonperseverative error (%) | 15.05 (7.86) | 10.2 (6.21) | 0.12 |
| Categories completed | 3.95 (2.27) | 5.3 (1.89) | 0.11 |

Data are shown as the mean (standard deviation) or n values.

Comparison of age, education year, BDI, total intracranial volume, total cerebellar volume between SCA group and ILOCA group, p-values were done with independent t-test. In the comparison of cognitive profiles between SCA group and ILOCA group, p-values were calculated with generalized linear model with age and sex as cofactors. Abbreviation, CA: Cerebellar ataxia, MMSE: K-Mini-mental status exams, MoCA: Montreal Cognitive Assessment, TMT: Trail Making Test, BDI: K-Beck’s Depression Inventory, SARA: scale for the assessment and rating of ataxia, SCA: Spinocerebellar ataxia, ILOCA: idiopathic late onset cerebellar ataxia.

**Supplementary Figure 1. Correlation of SARA scores with cognitive profiles in SCA2, other SCAs and ILOCA**


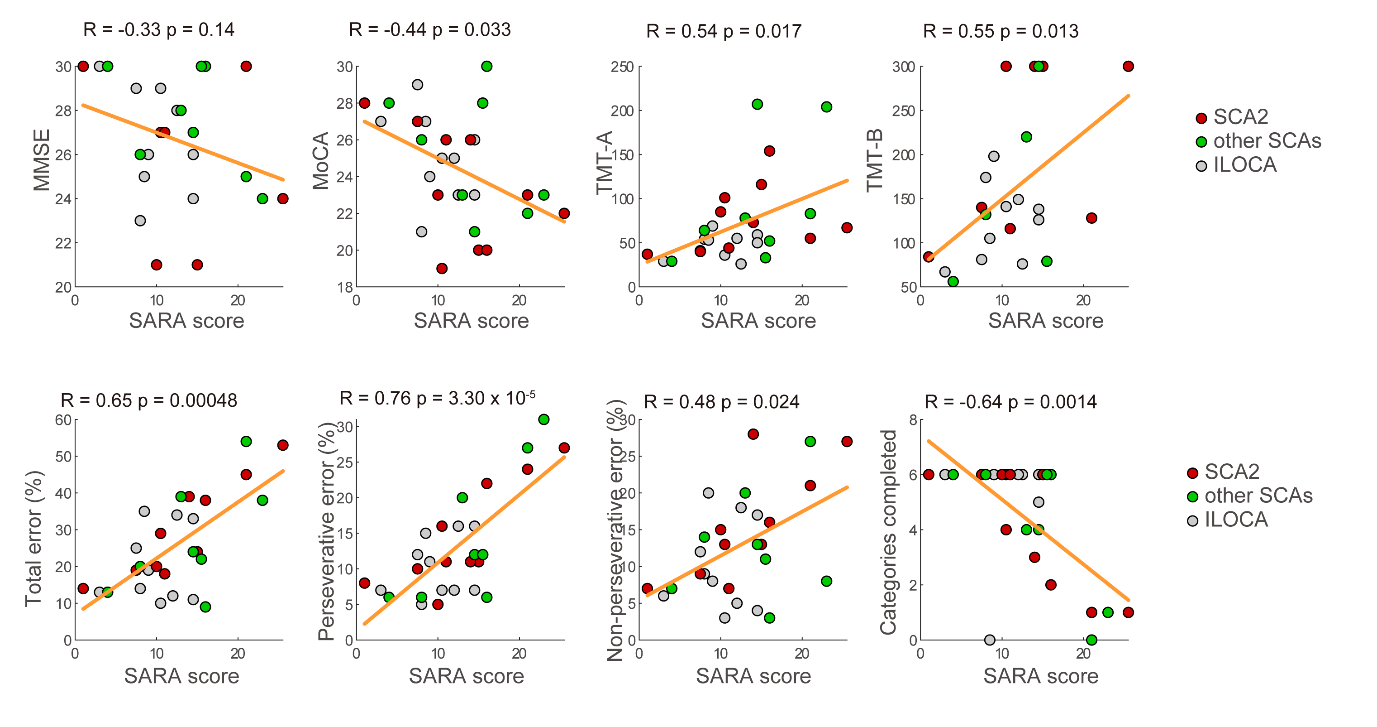


Scatter plots of the SARA score with cognitive profiles of MMSE (A), MoCA (B), TMT-A (C), TMT-B(D) and Wisconsin card sorting test scores (E-F) in the SCA group (SCA2: red, other SCAs: green, ILOCA: gray). The correlation was analyzed with partial correlation with age and sex as cofactors. Abbreviation, SCA: spinocerebellar ataxia, ILOCA: idiopathic late onset cerebellar ataxia, SARA: Scale for the Assessment and Rating of Ataxia, TMT: trail making test, MoCA: Montreal cognitive assessment battery and MMSE: Mini-mental status exam, WCST: Wisconsin Card sorting test. CA: Cerebellar ataxia.

**Supplementary Figure 2. Correlation of whole-brain volume with performance of WCST**


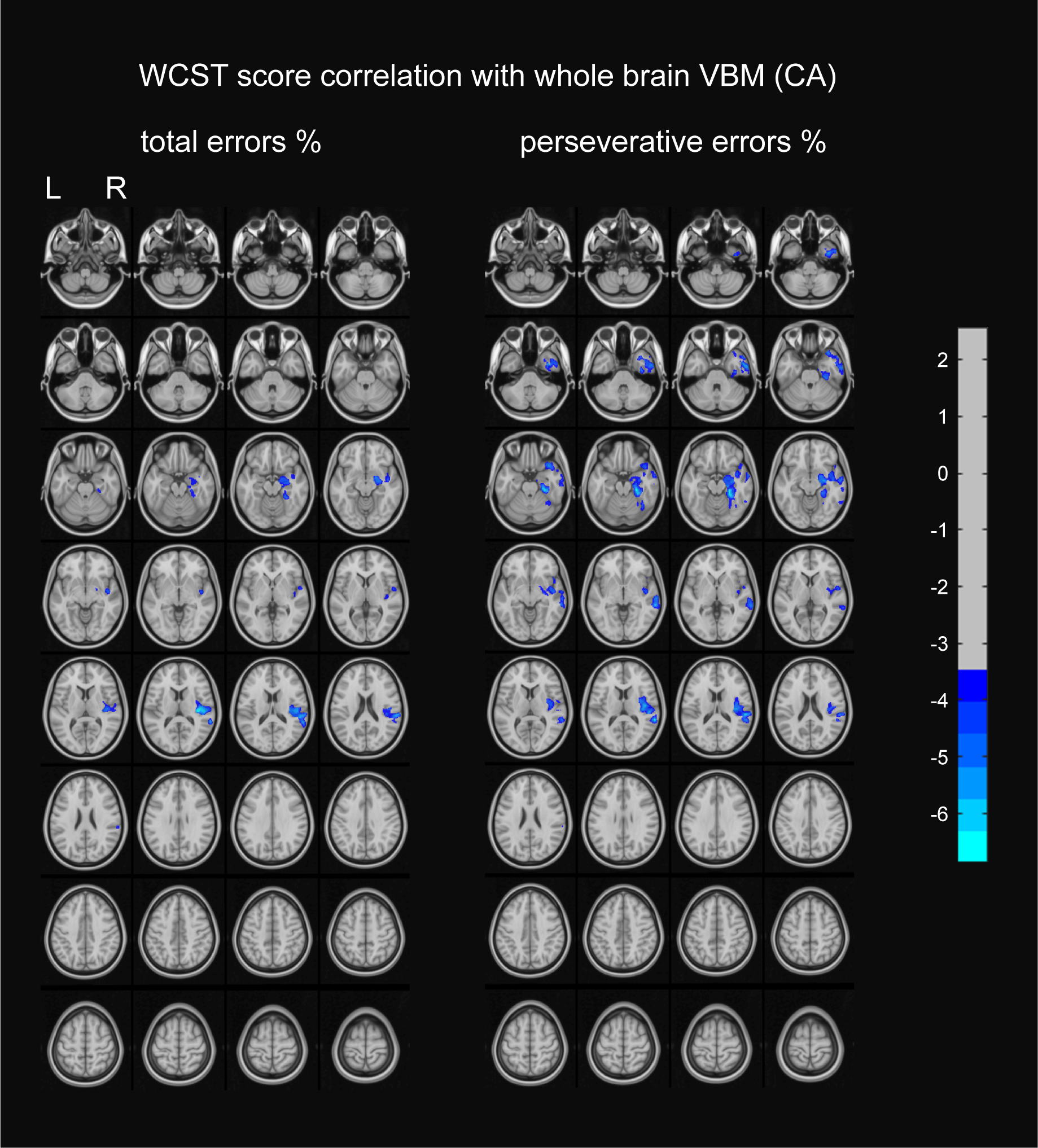


Whole brain correlation with total errors % and perseverative error % in Wisconsin card sorting test in CA group. Multiple corrections were done with the Gaussian random field method (voxel p < 0.001, cluster p < 0.01). Colorbar represents the T value. Abbreviation CA: Cerebellar ataxia, WCST: Wisconsin Card sorting test, , VBM: voxel-based morphometry, L: left, R: right

**Supplementary Figure 3. Correlation of cerebellar volume and whole-brain volume with WCST error in healthy controls**


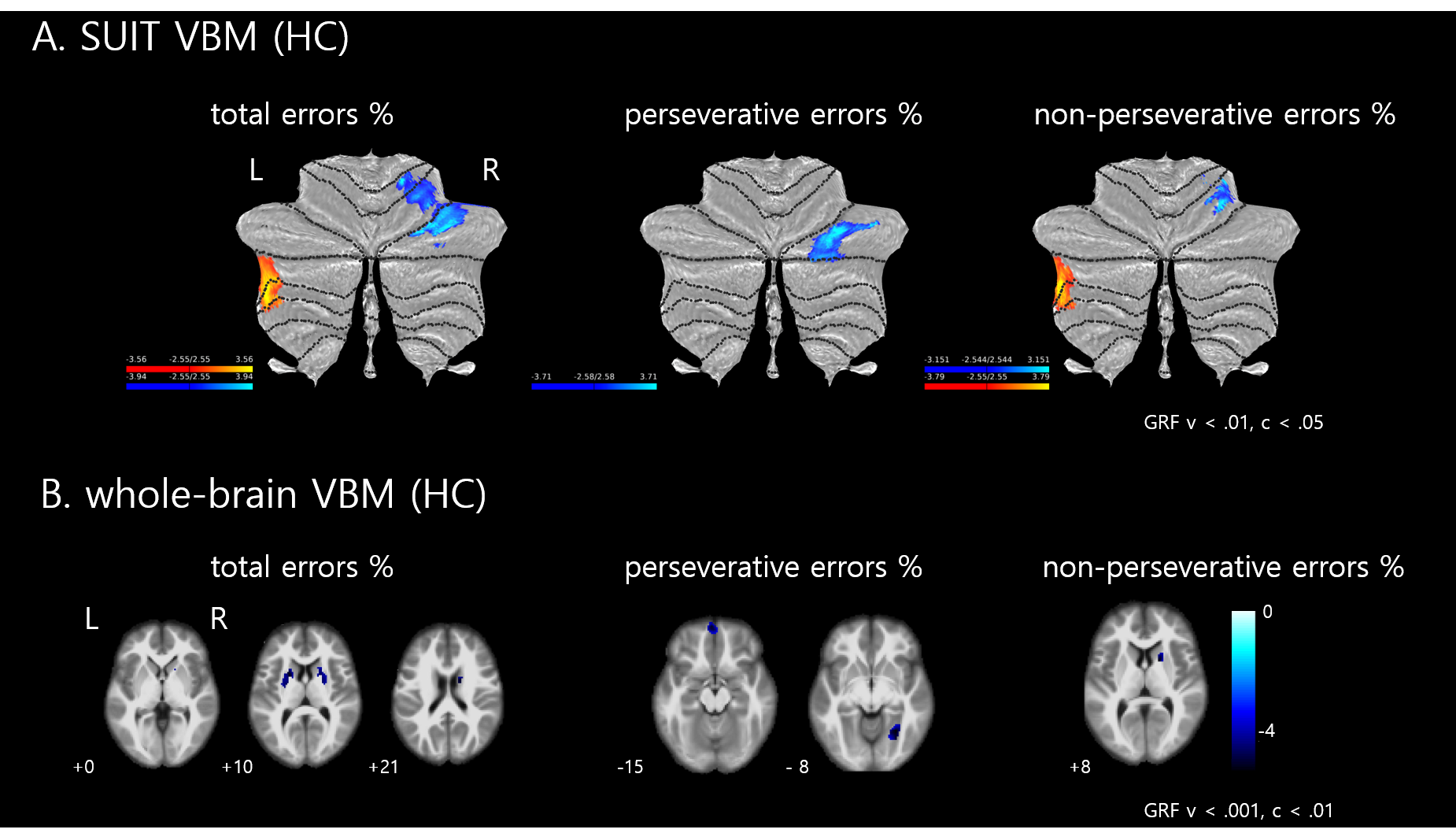


A. Cerebellar regional correlation with total error %, perseverative error % and non-perseverative error % in Wisconsin card sorting test in healthy controls. Multiple corrections were done with the Gaussian random field method (voxel p < 0.01, cluster p < 0.05). Colorbar represents T-value. B. Whole brain correlation with total errors %, perseverative error % and non-perseverative error % in Wisconsin card sorting test in healthy controls. Multiple corrections were done with the Gaussian random field method (voxel p < 0.001, cluster p < 0.01). Colorbar represents the T value. Abbreviation, HC: Healthy controls, SUIT: Spatially unbiased infratentorial, VBM: voxel-based morphometry, L: left, R: right.

**Supplementary Figure 4. Correlation of cerebellar volume with SARA score in CA patients**


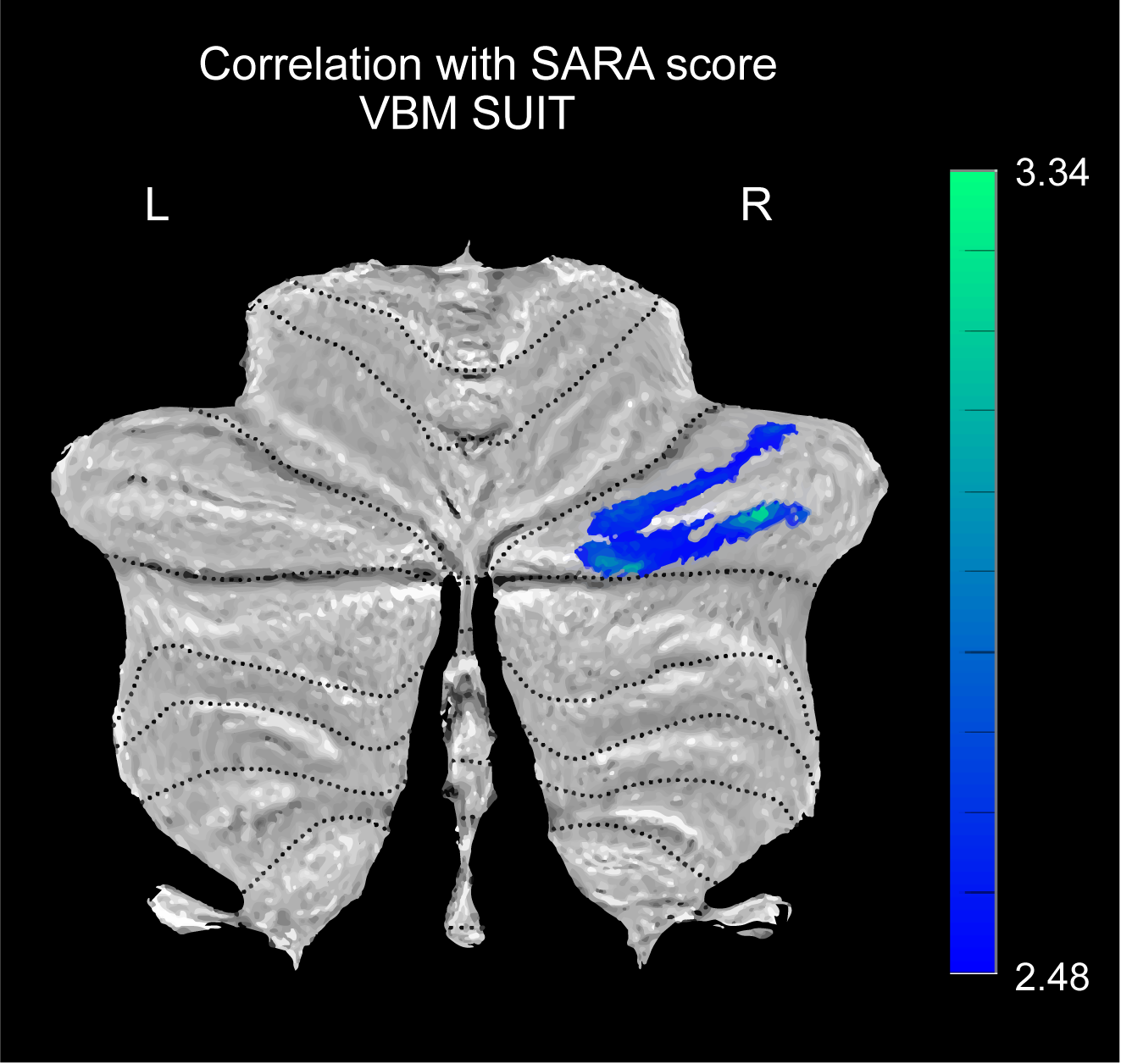


Cerebellar regional correlation with SARA scores in CA patients. Multiple corrections were done with the Gaussian random field method (voxel p < 0.05, cluster p < 0.05). Colorbar represents T-value. Abbreviation CA: Cerebellar ataxia, VBM: voxel-based morphometry, SUIT: Spatially unbiased infratentorial, L: left, R: right

**Supplementary Figure 5. Direct comparison of the whole brain functional connectivity of cognitive cerebellum between SCA group and healthy controls**


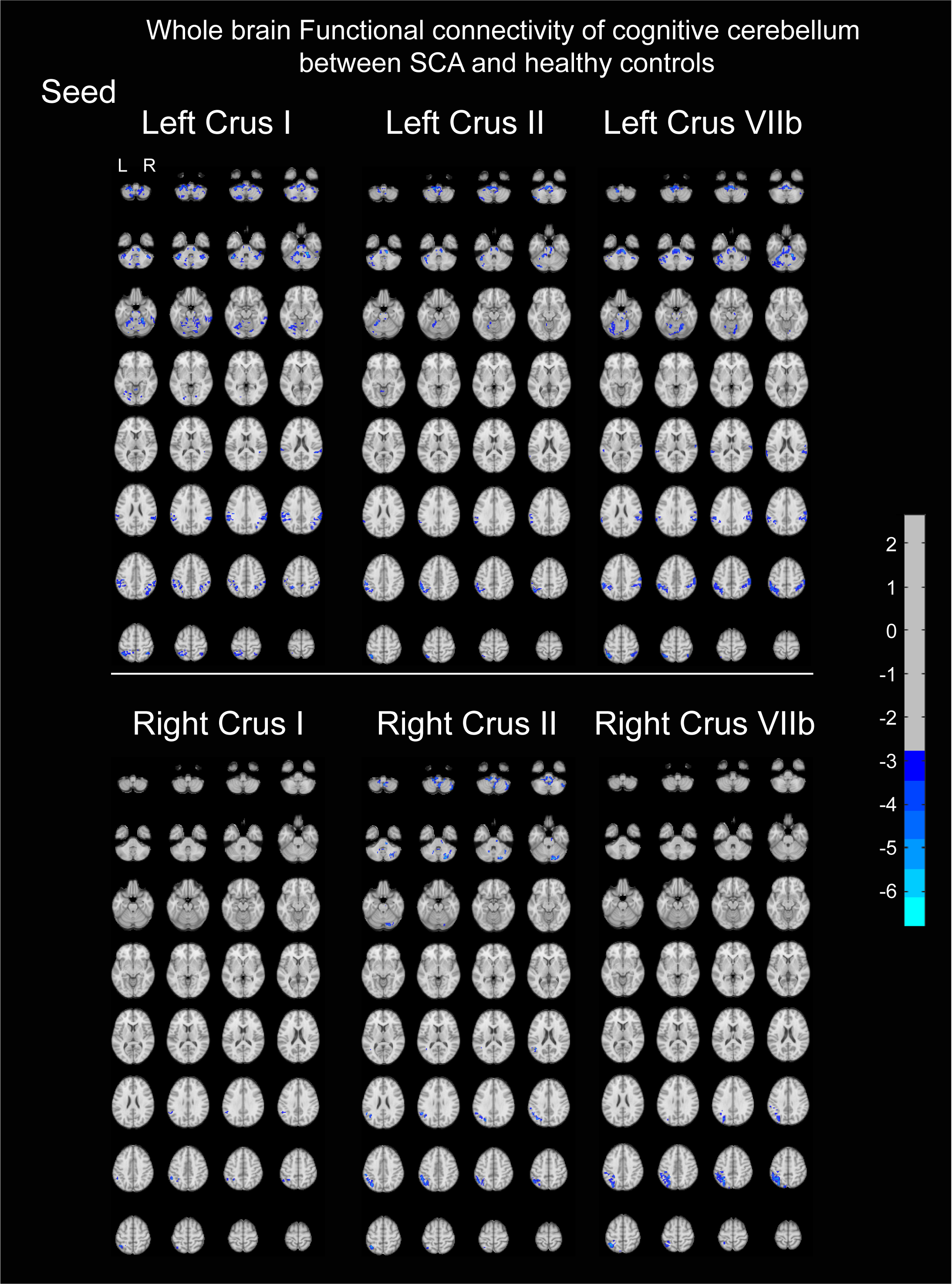


Comparison of seed-based whole-brain functional connectomics between CA and healthy control group. Multiple corrections were done with the Gaussian random field method (voxel p < 0.005, cluster p < 0.01). The color bar represents the T value.
